# Supplementary material for: Mechanisms governing the pioneering and redistribution capabilities of the non-classical pioneer PU.1
Source: Nat Commun. 2020 Jan 21;11:402. doi: 10.1038/s41467-019-13960-2 (PMC6972792; doi:10.1038/s41467-019-13960-2)
Supplement: Supplementary file 7 — Source data [file 41467_2019_13960_MOESM7_ESM.zip › Source_Data/Figure6/Figure6B_MotifScanOutput/ETS1_mutPU.1/homerResults/motif2.similar.html]

motif2

## Information for motif2

A
G
T
C
G
A
C
T
G
C
T
A
T
C
G
A
T
C
G
A
A
G
T
C
A
G
T
C
C
G
T
A
A
G
T
C
G
C
T
A
C
T
A
G
  
Reverse Opposite:  

G
A
T
C
C
G
A
T
A
C
T
G
G
C
A
T
A
C
T
G
A
C
T
G
A
G
C
T
A
G
C
T
C
G
A
T
C
T
A
G
T
C
A
G
  

|  |  |
| --- | --- |
| p-value: | 1e-1163 |
| log p-value: | -2.678e+03 |
| Information Content per bp: | 1.639 |
| Number of Target Sequences with motif | 5632.0 |
| Percentage of Target Sequences with motif | 41.76% |
| Number of Background Sequences with motif | 3857.3 |
| Percentage of Background Sequences with motif | 10.99% |
| Average Position of motif in Targets | 101.3 +/- 47.7bp |
| Average Position of motif in Background | 101.7 +/- 61.2bp |
| Strand Bias (log2 ratio + to - strand density) | -0.0 |
| Multiplicity (# of sites on avg that occur together) | 1.27 |
| Motif File: | file (matrix) reverse opposite |

### Similar de novo motifs found

|  |  |  |  |  |  |  |  |
| --- | --- | --- | --- | --- | --- | --- | --- |
| Rank | Match Score | Redundant Motif | P-value | log P-value | % of Targets | % of Background | Motif file |
| 1 | 0.996 | G A T C C G A T T A C G G A C T T C A G A C T G A G C T G A C T C G A T | 1e-1158 | -2667.451039 | 43.48% | 12.12% | motif file (matrix) |
| 2 | 0.989 | T C G A T A G C G C T A T C G A T C G A A G T C A G T C C T G A T A G C G C T A C T G A T G C A | 1e-1044 | -2404.156623 | 35.70% | 8.52% | motif file (matrix) |
| 3 | 0.984 | C G A T A T C G G C A T A C T G A C T G A C G T G A C T C G T A | 1e-1037 | -2388.980270 | 43.44% | 13.38% | motif file (matrix) |
| 4 | 0.941 | C A G T A T C G G A C T C T A G C T A G A C G T G A C T C G T A T C A G | 1e-965 | -2223.799177 | 61.50% | 28.49% | motif file (matrix) |
| 5 | 0.919 | G C T A C T A G T C G A A G T C A G T C C T G A A T G C G C T A | 1e-925 | -2132.068628 | 55.88% | 24.23% | motif file (matrix) |
| 6 | 0.971 | A C G T A C T G A G C T A C T G A C T G A C G T G A C T | 1e-856 | -1972.806150 | 34.75% | 9.81% | motif file (matrix) |
| 7 | 0.939 | A C T G C A G T A C T G C A T G A C T G G A C T C A G T A C T G A C G T A C T G A C T G A C G T A G C T | 1e-826 | -1902.284254 | 38.27% | 12.40% | motif file (matrix) |
| 8 | 0.944 | G T C A T C G A T G C A A G T C A G T C C G T A A G T C G C T A C T A G T G A C G T A C G T C A G T C A T A G C | 1e-796 | -1833.377453 | 40.68% | 14.42% | motif file (matrix) |
| 9 | 0.922 | C T A G G C A T T C A G C T A G A C G T C A G T C G T A | 1e-666 | -1533.848056 | 55.39% | 28.18% | motif file (matrix) |
| 10 | 0.900 | A G C T A C G T A C T G A G C T A C T G A C T G A G C T | 1e-609 | -1404.458026 | 40.52% | 17.01% | motif file (matrix) |
| 11 | 0.868 | A G T C C G T A T C G A A G T C G A T C C T G A A G T C G C T A C T A G T C G A | 1e-606 | -1397.173344 | 42.24% | 18.37% | motif file (matrix) |
| 12 | 0.808 | A G C T C A G T A C T G A G T C C T A G A C T G G A C T G A C T C A G T | 1e-602 | -1386.429108 | 41.46% | 17.86% | motif file (matrix) |
| 13 | 0.832 | C T G A C T G A G A T C A G T C C T G A A G T C T C G A G C A T | 1e-551 | -1268.861528 | 33.50% | 12.85% | motif file (matrix) |
| 14 | 0.895 | C T A G T C A G C G T A T C G A C T G A A G T C A G T C C G T A G A T C C G T A T A C G C G T A A C T G | 1e-467 | -1076.897234 | 19.70% | 5.26% | motif file (matrix) |
| 15 | 0.763 | T C G A T G A C G C T A C T A G T C A G C G T A C T G A T C G A A T G C G T A C G T C A T A G C G T C A T C G A | 1e-380 | -876.834614 | 27.04% | 11.06% | motif file (matrix) |
| 16 | 0.764 | C A T G A T G C A G T C A G T C C G T A A G T C C G T A | 1e-272 | -626.593832 | 42.83% | 26.07% | motif file (matrix) |
| 17 | 0.759 | C A G T G A T C C G A T C T A G G A C T T C A G A C T G A C G T | 1e-218 | -502.340430 | 20.25% | 9.30% | motif file (matrix) |
| 18 | 0.775 | A C G T A C G T A C G T A C T G A G C T A C T G A C T G A C G T A T C G | 1e-138 | -319.001477 | 4.16% | 0.68% | motif file (matrix) |
| 19 | 0.823 | C A G T A T C G G C A T A C T G A C T G A C G T C A G T T C G A T C G A T A G C G T A C G C T A | 1e-123 | -284.029098 | 4.93% | 1.16% | motif file (matrix) |
| 20 | 0.850 | T C A G T G A C C G T A A T C G C G A T A C T G C G A T A C T G A T C G A C G T A C G T C G T A T C A G C T G A | 1e-78 | -180.805093 | 2.95% | 0.65% | motif file (matrix) |
